# Fractured Jaw

**Published:** 1889-09

**Authors:** Louis P. Hall


### Fractured Jaw.

BY LOUIS P. HALL, D.D.S.

Though it is probable that not one dentist in twenty ever has, in his own practice, a case of fractured jaw, it is nevertheless quite an important part of his education to learn and master all the particular points in such a case, at least so far as the theoretical knowledge is to be obtained.

How many cases of deformed faces do we see in our every day life, and as dentists we can but wonder how they were caused, or how they might have been prevented had some skilled surgeon or dentist or both, been called in time.

In ordinary cases of simple fracture the physician is usually able to adjust the parts and all will be well, but in the more complicated or comminuted fractures, the assistance of the dentist is most needed, and will hereafter be most often called upon.

The study of medicine and surgery in all its branches is becoming more and more specialized every year, and to the dentist studying continually the mouth and the tissues and parts about it, must come eventually such cases as make up the subject of this short paper.

The most frequent source of fracture is gunshot wounds. In these the fracture is usually at the place of contact because the bones about the head are more friable and less dense in structure, but fractures may occur from falls, flying debris and other causes. Other conditions being alike fracture is more apt to occur in those persons having the most inorganic material in the bone structure.

The majority of fractures, other than gunshot wounds, are in the body of the jaw, as from falls or blows on the symphysis the force is usually transmitted to a more friable part. Frank Hamilton states that 50 out of 53 cases examined by him were fractures of the body, dislocation being more apt to occur before fracture of the ramii.

The signs and symptoms are one or more of the following: Deformity—unless impacted; and the deformity may be either that the chin points to the side of fracture caused by contracting muscles drawing the parts past, broken pieces of jaw drawn out of place, or fragments of jaw may protrude through the tissues.

Under certain conditions increased motion, crepitus of fractured parts, laceration of gums, salivation and pain, though these last two may occur from other causes and are not to be relied upon.

The deformity may not be great and only one or more of these signs be present at once. The inferior dental nerve and arteries are seldom ruptured, though when an artery is ruptured the hemorrhage is quite severe and often very difficult to control.

In simple cases, replace the parts as nearly as possible taking care that no loose fragments remain to obstruct the union; hold the parts firmly in place by means of a suitable bandage, preferably the four tailed bandage, and keep the patient quiet. In the more comminuted cases, the wholly separated portions and any loosened teeth, especially if they are at the point of fracture and tend to obstruct union had best be removed. Other parts may be held more firmly together by means of wires passed through holes drilled in ends or by means of ligatures about the teeth. In all cases the patient should be instructed to sleep in such a position that as little as possible of the pus be swallowed. Disinfectants should be used and other precautions followed as in other fractures.

Having adjusted the fragments as nearly as possible to their normal position an impresssion should be taken of each arch and models made from these. Now cut model of broken jaw in such sections as with the least arrangement it can be best made to articulate with the model of whole jaw. Take an impression of the sections as thus arranged and pour up a new model and upon this construct a coffin plate, imbedding in either side a strong u-shaped wire so that the free ends may extend outside and back from the mouth. Place the plate on the jaw and over the teeth corresponding to the sockets. If the fracture has been of some standing it may be necessary to divide certain portions of the jaw as the model was divided for better articulation, and then crowd the teeth well into the plate, and by means of the external wires and convenient support for chin, the jaw may be held firmly in place while nature is doing her share in reconstructing the jaw. Foods must be of a liquid nature and taken through an opening left for that purpose in the plate which should be worn as long as possible at a time without being removed. It may be necessary to construct several such plates, differing more or less as the case proceeds. Fractures will usually heal in from four to six weeks.

ANTISEPTIC MOUTH WASH.—Galippe and Malasez give, in *Nouveaux Remedes* No. 55, the following formula for an antiseptic mouth wash which has been found satisfactory by them:

| | | |
|---|---|---|
| Alchohol | parts | 370 |
| Carbolic Acid | " | 10 |
| Thymol | " | 5 |
| Oil of peppermint | " | 15 |
| Tincture of anise | " | 100 |

This mixture, which may be colored with some tincture of cochineal, should be employed morning and evening, and the mouth rinsed out at the same time with a weak solution of boric acid.

